# Constructing modular and universal single molecule tension sensor using protein G to study mechano-sensitive receptors

**DOI:** 10.1038/srep21584

**Published:** 2016-02-15

**Authors:** Xuefeng Wang, Zainab Rahil, Isaac T. S. Li, Farhan Chowdhury, Deborah E. Leckband, Yann R. Chemla, Taekjip Ha

**Affiliations:** 1 Department of Physics, Center for the Physics of Living Cells, University of Illinois at Urbana-Champaign, Urbana, Illinois 61801, USA; 2 Institute for Genomic Biology, University of Illinois at Urbana-Champaign, Urbana, Illinois 61801, USA; 3 Department of Physics and Astronomy, Iowa State University, Ames, Iowa 50011, USA; 4 Department of Chemistry, University of Illinois at Urbana-Champaign, Urbana, Illinois 61801, USA; 5 Department of Chemistry, University of British Columbia Okanagan, Kelowna, BC, V1V1V7, Canada; 6 Mechanical Engineering and Energy Processes, Southern Illinois University, Carbondale, Illinois 62901, USA; 7 Howard Hughes Medical Institute, Baltimore, MD, 21218, USA; 8 Department of Biophysics & Biophysical Chemistry, Johns Hopkins University, Baltimore, MD 21205, USA; 9 Department of Biomedical Engineering, Johns Hopkins University, Baltimore, MD 21205, USA

## Abstract

Recently a variety of molecular force sensors have been developed to study cellular forces acting through single mechano-sensitive receptors. A common strategy adopted is to attach ligand molecules on a surface through engineered molecular tethers which report cell-exerted tension on receptor-ligand bonds. This approach generally requires chemical conjugation of the ligand to the force reporting tether which can be time-consuming and labor-intensive. Moreover, ligand-tether conjugation can severely reduce the activity of protein ligands. To address this problem, we developed a Protein G (ProG)-based force sensor in which force-reporting tethers are conjugated to ProG instead of ligands. A recombinant ligand fused with IgG-Fc is conveniently assembled with the force sensor through ProG:Fc binding, therefore avoiding ligand conjugation and purification processes. Using this approach, we determined that molecular tension on E-cadherin is lower than dsDNA unzipping force (nominal value: 12 pN) during initial cadherin-mediated cell adhesion, followed by an escalation to forces higher than 43 pN (nominal value). This approach is highly modular and potentially universal as we demonstrate using two additional receptor-ligand interactions, P-selectin & PSGL-1 and Notch & DLL1.

Mammalian cells mechanically interact with neighboring cells and the extracellular matrix (ECM) through a variety of cell surface receptors such as integrins^1^, cadherin^2^, P-selectin^3^, Notch receptor^4^, Eph receptor^5^, etc. Forces on these receptors are important for sensing the environment, activating receptor-mediated signaling pathways^6^,^7^,^8^ and regulating associated cellular functions. In recent years, methods have been developed to measure the molecular forces on single receptors. One commonly adopted strategy is to conjugate a force-reporting tether to a ligand molecule and immobilize the ligand on a surface through the tether. Cognate cell membrane receptors bind to the ligands and transmit tension to the tethers which then report the molecular tension by conformational changes or rupture. Elastic PEG linear polymer, hairpin DNA, duplex DNA, and spider silk peptide decorated with a FRET (fluorescence resonance energy transfer) pair have been adopted as the force-reporting tethers^9^,^10^,^11^,^12^. We reported a double-stranded DNA-based force sensor named tension gauge tether (TGT) which is a rupturable tether with a tunable tension tolerance (*T*_tol_)^13^. When TGTs conjugated with a RGDfK peptide targeting integrin α_V_β_3_ were presented to the cells, cells adhered poorly for *T*_tol_ ≤ 33 pN because the TGTs were ruptured by cellular forces exerted through single integrins while they adhered stably for *T*_tol_ ≥ 43 pN. This tension threshold for adhesion was universal for a variety of cancerous and noncancer cell types^14^ and was in operation as early as five minutes after cell plating^13^. Therefore, we concluded that the peak force across a single integrin-ligand bond during initial cell adhesion is about 40 pN. In a subsequent study, we used the TGT platform to show that the adhered cells spread more extensively as *T*_tol_ increases beyond 43 pN and that it is the molecular tension, not the molecular stiffness, that dictates the degree of cell spreading^14^. We further demonstrated that the molecular tension on clustered integrins in focal adhesions can go beyond 54 pN^15^.

These approaches of linking ligands to tension sensing tethers have been applied mainly to integrin studies because the widely adopted integrin ligand, RGD peptides^16^, tolerate ligand-tether conjugation and purification processes, presumably because these ligands are short peptides and have no secondary structures. However, for many other potentially mechano-sensitive receptors such as cadherin, Eph receptor, Notch receptor and P-selectin, both these receptors and their ligands are proteins with considerably higher molecular weight and potentially delicate structures. For example, we conjugated human epithelial cadherin (Ecad) to a DNA strand in an attempt to study cadherin tension, but observed that the Ecad activity is significantly reduced after conjugation (Fig. 1A). DLD-1 cells expressing Ecad adhered and spread normally on the surface with physically adsorbed unconjugated Ecad whereas the same cells adhered less and spread poorly on the surface with physically adsorbed Ecad-ssDNA conjugates. Both surfaces were coated with Ecad at a saturating incubation concentration of 100 μg/ml for 1 hour. To retain ligand activity and also to provide a universal and modular force sensor for many types of receptors, here we introduce Protein G (ProG)-based TGT.

Our strategy is to conjugate a force reporting tether to a ProG instead of the ligand itself. ProG binds to the Fc domain of immunoglobulin G (IgG) with high affinity and has been extensively used in IgG purification^17^. Fc-fused recombinant ligands targeting cadherins, Ephrin receptor, Notch receptor and other receptors are widely available commercially and in many research labs^18^,^19^. Fc-fused P-selectin is also available and has been used to study leukocyte rolling mediated by P-selectin and PSGL-1 (P-selectin glycoprotein ligand-1) interaction^20^. In principle, ProG binds to Fc region of Fc-fused ligands and should not interfere with the ligand activity. We confirmed that Ecad activity was not reduced after binding to ProG as DLD-1 cells adhered and spread normally on a glass surface physically adsorbed with Ecad-Fc:ProG (SFig. 1). We also confirmed that Ecad activity was retained after being immobilized through ProG-biotin on pegylated glass surface which is the main experimental platform we used in this article (SFig. 2). Because native ProG has an albumin binding domain and a membrane binding domain which can cause nonspecific binding^21^, we used a ProG fragment, amino acids 190–384, which contains the IgG-Fc binding domain (ab49807, Abcam). We conjugated this ProG to our double stranded DNA-based TGT (Fig. 1B) using a bifunctional linker that targets a thiol on DNA and one of lysines on ProG as detailed in the Methods section. A biotin on TGT is used to immobilize a ligand on the surface in the format of ligand-Fc:ProG-TGT-biotin:neutravidin:biotin-surface.

## Results and Discussion

### T_tol_ values of TGTs used in this article are nominal values

**Figure 1:**
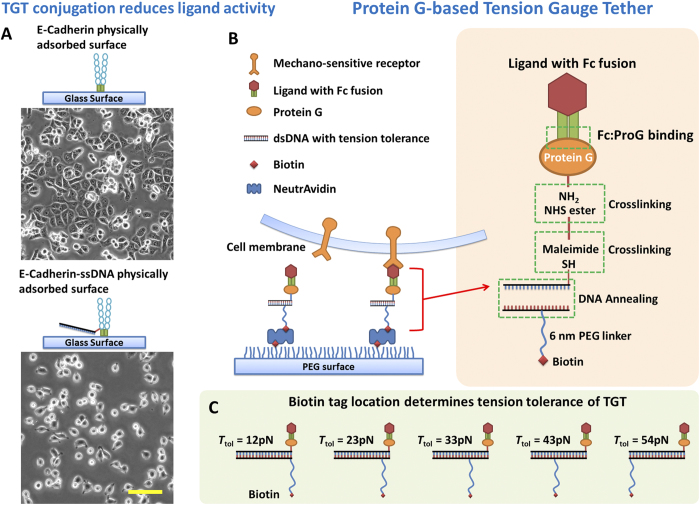
ProG-based Tension Gauge Tether. (**A**) Direct DNA conjugation on cadherin molecules reduced cadherin activity, leading to poorer DLD-1 cell adhesion and spreading. (**B**) The schematics of ligand immobilization through ProG-based TGT. Recombinant ligands with IgG-Fc fusion are assembled with ProG-TGT and immobilized on a glass surface passivated with polyethylene glycol (PEG). (**C**) Biotin tag was used to immobilize the ligand-TGT constructs. Biotin tag location on the dsDNA determines the tension tolerance *T*_tol_ of TGTs. Note that the *T*_tol_ values used in this article are nominal because the time scale of cellular force application is unknown. Scale bar: 100 μm.

The *T*_tol_ value set of 12, 23, 33, 43 and 54 pN used in this article was predicted by de Gennes model with parameters derived from calibrated DNA shear force^22^. A recent improved model gives a new *T*_tol_ set of 14, 16, 19, 30 and 54 pN by taking experimental temperature and time scale into consideration^23^. These two models have insignificant deviation at fully unzipping and fully shear configurations but do have considerable differences for intermediate *T*_tol_ values. In addition, it is known that the critical force to rupture a molecular bond is dependent on the rate of force increase. Therefore, to apply the pre-calibrated *T*_tol_ values for cellular force measurement, the time scale of cellular force on a receptor-ligand bond should not significantly deviate from the time scale of *T*_tol_ calibration. The magnetic tweezers experiments that calibrated DNA rupture force used a time scale of 2 seconds by increasing the force incrementally after 2 seconds of constant force until DNA ruptured^22^. However the cellular rates of force increase across cadherins are unknown to our knowledge. Therefore, these *T*_tol_ values may be different from true values and they should be considered nominal in this article. Here we continue to use the *T*_tol_ values derived from de Gennes model based on 2 second calibration time for the convenient comparison with previous experimental results. We previously estimated that the relevant time scale of force application through single integrins in the context of motile focal adhesions is from seconds to minutes^23^. *T*_tol_ values have insignificant variation (<10%) in this time range^22^,^23^. The monotonic increase of *T*_tol_ as TGT geometry tuned from unzipping mode to shear mode (Fig. 1C) should be unchanged at different time scales, allowing us to explore cellular forces on single receptors without the knowledge of the time scale of force application.

### Binding test of ProG-TGT based molecular force senor

**Figure 2:**
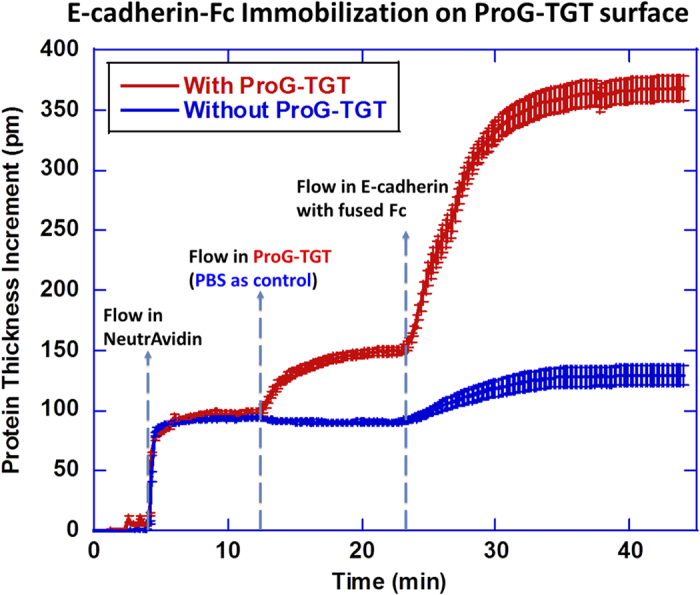
Micro-ring resonator assay for immobilization of Fc-fused Ecad through ProG-TGT on polymer-passivated glass surface. Concentration of reagents: 200 μg/mL neutravidin, 0.05 μM ProG-TGT and 50 μg/mL Ecad. Blue curve is the control run in which PBS buffer was flowed across the surface instead of ProG-TGT solution.

We confirmed ProG-TGT’s ability to bind to Fc using the micro-ring resonator assay^24^. 200 μg/ml neutravidin, 0.05 μM ProG-TGT and 50 μg/ml Ecad-Fc (648-EC-100, R&D systems) solutions were successively flowed over a polymer-passivated surface presenting biotin. The binding of these molecules on the surface increased molecular film thickness which was detected by the micro-ring resonator (Fig. 2). Adding ProG-TGT to the neutravidin surface caused an increase of 56 pm in film thickness. A subsequent addition of Ecad-Fc gave rise to a further 237 pm thickness increase. A negative control skipping the ProG-TGT step gave a six-fold smaller thickness increase of 38 nm. Because ProG-TGT has a molecular weight of 36 kDa and Ecad-Fc has a molecular weight of 175 kDa, we infer that the final molar ratio between ProG-TGT and Ecad is 1:0.87, indicating nearly stoichiometric capture of Ecad-Fc by ProG-TGT.

We then used Ecad-Fc:ProG-TGT to study cell adhesion mediated by cadherins. To function in cell-cell interactions which are important for embryo development and tissue maintenance^2^, cadherins act both as a receptor and as a ligand^25^. To mimic cadherin-cadherin interactions during cell-cell adhesion, we immobilized Ecad on a flat and solid surface for the convenience of surface chemistry and imaging^26^,^27^,^28^. We mixed Ecad-Fc with ProG-TGT at molar ratio 1:1 and final concentration of 1 μM in Dulbecco’s Phosphate-Buffered Saline (DPBS with calcium and magnesium) buffer and kept the mixture at 4 °C for 30 min. The final construct Ecad-Fc:ProG-TGT, which we call Ecad-TGT, was then immobilized through biotin on a polymer-passivated glass surface (Fig. 1B). The biotin location on the TGT determines *T*_tol_^13^. In the final construct, we used 6 nm long PEG12 linker for TGT immobilization to overcome the steric hindrance caused by large-sized ligand molecules (SFig. 3). Such steric hindrance would give rise to lower surface densities for intermediate TGTs and potentially lead to a bias of force measurement if the original 2 nm PEG 2 linker was used (details included in supplementary information).

**Figure 3:**
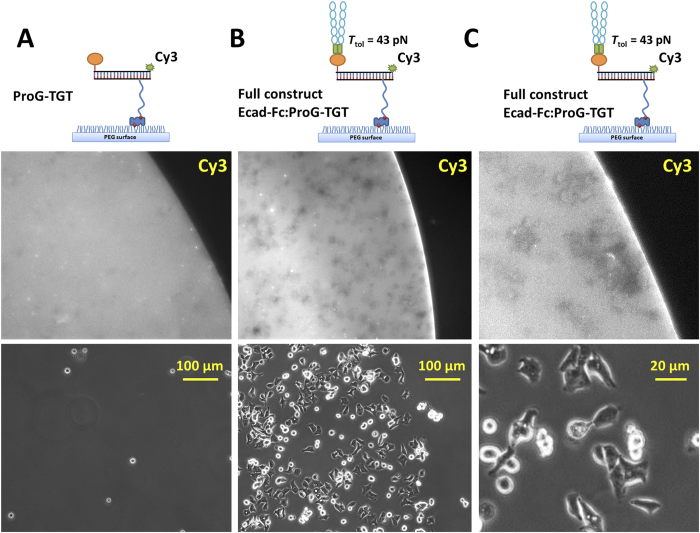
DLD-1 cells specifically adhere on Ecad-Fc:ProG-TGT coated surface. (**A**) Cells did not adhere on ProG-TGT grafted surface (no Ecad-Fc). Middle panel shows a fluorescence image of Cy3 conjugated to ProG-TGT. The bright area marks the ProG-TGT coated region. Bottom panel shows a phase contrast image of the same area. (**B**) Cells specifically adhered on Ecad-Fc:ProG-TGT coated surface marked by strong fluorescence. Fluorescence loss (gray patches in TGT grafted area) underneath the cells suggests that cells caused TGT rupture. (**C**) Under higher magnification, it is evident that gray patches of fluorescence loss are co-localized with cells, confirming that TGTs were ruptured by DLD-1 cells.

In order to test the specificity of cell adhesion on the Ecad-TGT surface, we compared DLD-1 cell adhesion on ProG-TGT (43 pN) surface with and without Ecad-Fc incubation (Fig. 3A). Surface preparation and cell plating procedures are detailed in Methods. The surface density of adhered cells was 386/mm^2^ on the Ecad-TGT surface with Ecad-Fc compared to only 8/mm^2^ on the control surface, demonstrating that the cell adhesion is mediated by Ecad, not ProG-TGT. Simultaneous fluorescence imaging of Cy3 fluorophore conjugated to the ProG-TGT showed that cells adhered only on the region of the surface coated with Ecad-TGT, suggesting minimal non-specific cell adhesion. Loss of fluorescence in dark patches under the cells suggests that cells caused TGT rupture (Fig. 3B,C), likely by exerting forces through Ecad-Ecad bonds, and that the ProG:Fc bond is strong enough to transmit the tension needed to rupture 43 pN TGT. Cellular forces on single integrin receptors within focal adhesions can even remove biotin from streptavidin^29^ and therefore the loss of fluorescence for the Ecad-TGT shown above could be due to biotin-streptavidin rupture instead of 43 pN TGT rupture. We consider this scenario unlikely because we previously showed by two color imaging of differently labeled DNA strands that, for 54 pN TGT, over 95% of rupture occurs at the DNA^15^. Overall, our data show that 43 pN TGT can support DLD-1 cell adhesion through Ecad-Ecad interaction and that DLD-1 cells can apply >43 pN tension through single Ecad-Ecad bonds during or after the establishment of stable cell adhesion.

### Molecular tension on cadherins measured by ProG-TGT

**Figure 4:**
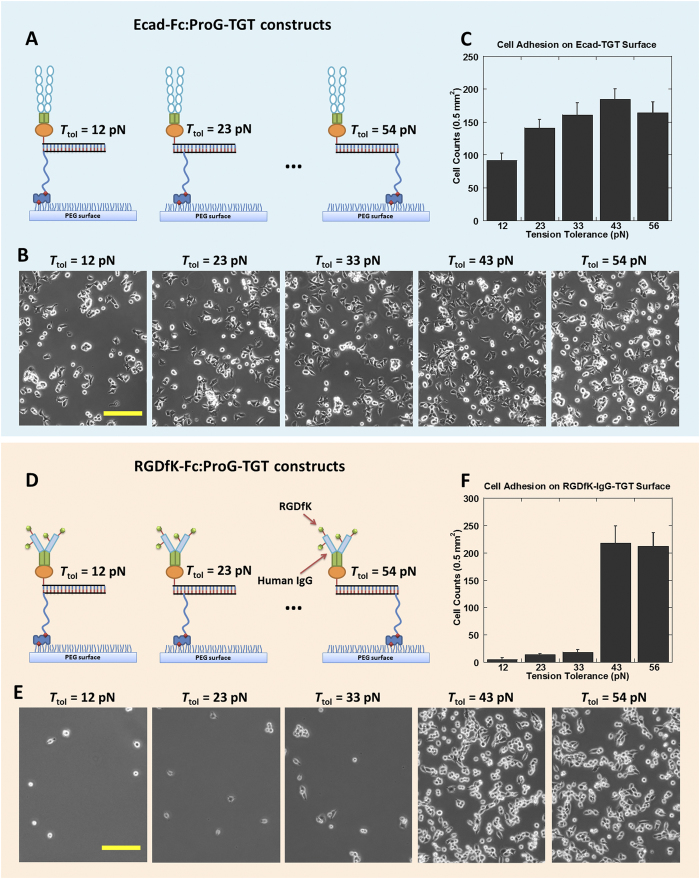
Molecular tension measurement on cadherin and integrin. (**A**) Schematics of Ecad immobilization through 12–54 pN ProG-based TGTs for cadherin tension measurement. (**B**) DLD-1 cells adhered on all 12~54 pN Ecad-Fc:ProG-TGT coated surfaces. (**C**) Cell adhesion density vs. *T*_tol_. (**D**) Schematics of RGDfK-Fc immobilization through 12–54 pN ProG-based TGTs for integrin tension measurement. (**E**) Phase-contrast images of CHO-K1 cell adhesion on 12~54 pN RGDfK-Fc:ProG-TGT coated surfaces. (**F**) Cell adhesion density vs. *T*_tol_. Scale bar: 100 μm.

To determine the single molecular forces required for cadherin-mediated cell adhesion, we repeated the cell adhesion experiment for five different TGTs with *T*_tol_ values ranging from 12 to 54 pN (Fig. 4A–C). DLD-1 cells were able to adhere on all five TGT surfaces with similar cell densities, indicating that the peak tension across a single Ecad-Ecad bond required for initial cell adhesion is below 12 pN. For comparison, we showed previously that the peak tension across a single integrin-RDGfK bond during integrin-mediated cell adhesion is about 40 pN and the peak tension across a single Notch-Delta bond required for Notch activation is below 12 pN^13^.

This initial study of cadherin tension yielded seemingly contradictory results. Fluorescence imaging revealed that Ecad tension can rupture TGT with *T*_tol_ = 43 pN. In contrast, cell adhesion assay demonstrated that DLD-1 cells were able to adhere on TGT with *T*_tol_ = 12 pN, suggesting that Ecad tension remains below 12 pN during initial adhesion. This result is reminiscent of a previous Ecad study that reported multi-level cadherin tensions^30^. Dunn and colleagues found that Ecad on cell membrane was constitutively under tension, and cell stretching after cell-cell contact (here cell-substratum contact) further increased Ecad tension. Therefore, we propose that two levels of cadherin tension contribute to our observations. During the initial cadherin-mediated cell adhesion, cadherin tension is lower than 12 pN. However, after stable cell adhesion, some cadherin tension increased to a much higher level which ruptures TGT with *T*_tol_ = 43 pN.

To further validate ProG-based force sensor, we used ProG-TGT to measure the peak tension across single integrins during integrin-mediated cell adhesion^13^. We conjugated RGDfK peptide to human IgG (See Method). IgG-RGDfK provides both the integrin ligand and the Fc domain for surface immobilization through ProG-TGT (Fig. 4D). 2 μM IgG-RGDfK was mixed with 2 μM ProG-TGT at 1:1 volume ratio and incubated at 4 °C for 30 min and was spotted on a biotinylated and polymer-passivated glass coverslip. CHO-K1 cells adhered poorly on ≤33 pN TGT and below, but adhered well on 43 and 54 pN TGT surfaces, indicating that integrin peak force is in the range of 33~43 pN (Fig. 4E,F), consistent with previous measurement using the regular RGDfK-TGT where RGDfK is directly conjugated to a DNA strand. This assay further suggests that ProG-Fc bond is strong enough not to act as the weak link in cell adhesion measurements.

### Molecular tension range on Notch receptor and P-selectin determined by ProG-TGT

**Figure 5:**
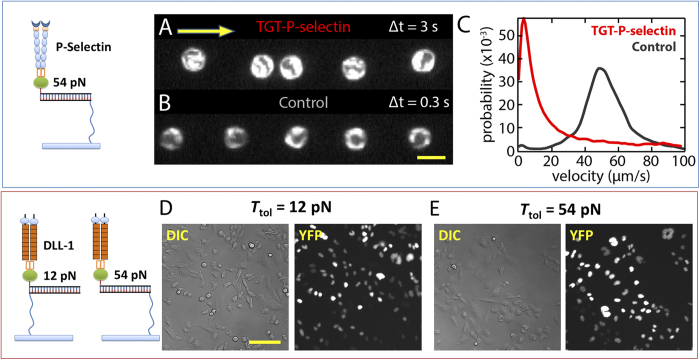
ProG-TGT for P-selectin and Notch receptor. (**A**) P-selectin-Fc:ProG-TGTs were assembled and immobilized on the inner surface of a flow chamber where leukocytes were flowed through. A single leukocyte location was captured by consecutive dark field imaging with 3 sec time interval. Yellow arrow indicates flow direction. (**B**) Single leukocyte location with 0.3 sec time interval on control surface without P-selectin coating. Scale bar: 20 μm. (**C**) Velocity distribution. HL-60 cells attached onto P-selectin-Fc:ProG-TGTs and show rolling behavior. This resulted in a cell moving rate significantly lower than the rate on control surface. (**D**) DLL1-Fc:ProG-TGTs were assembled and immobilized on glass surface where Notch activation was tested. Notch receptors were activated on both *T*_tol_ = 12 pN surface and *T*_tol_ = 54 pN surface after incubation for two days. Notch activation was reported by H2B-YFP expression in CHO-K1 cell nucleus. Scale bar: 50 μm.

Next, we tested if the ProG-TGT approach can also be used to examine single bonds between P-selectin and PSGL-1. During inflammation, P-selectin on the endothelial cell membrane binds to PSGL-1 expressed on leukocyte membrane. These bonds pull down leukocytes from blood flow and mediate leukocyte rolling^3^,^31^, an important step of leukocyte extravasation. We prepared a flow chamber where one inner surface was coated with P-selectin-Fc:ProG-TGT with *T*_tol_ = 54 pN, and leukocytes (HL-60 cells) were flowed across the surface (Fig. 5A). Leukocytes made transient contacts with the surface and started rolling, greatly slowing down their migration in the flow direction. In contrast, on the control surface coated with ProG-TGT without P-selectin, leukocytes moved at the same rate as the flow rate of culture medium, showing no sign of rolling on the surface. This experiment suggests that P-selectin molecules immobilized by ProG-TGT were active and the tension across single P-selectin/PSGL-1 bonds required for leukocytes rolling is lower than 54 pN.

Finally, we tested if Notch signaling activation can be studied using the ProG-TGT approach. Notch receptors on the cell membrane interact with Notch ligands presented by cell membranes of adjacent cells, and the signaling trigger by such interactions is important for cell-cell communication, cell differentiation and cell fate determination^32^. Recent research suggests that Notch activation requires tension applied to the Notch-ligand bond^6^,^19^,^33^. We assembled DLL-1-Fc:ProG-TGTs and immobilized them on a glass surface. DLL-1 (delta-like ligand 1) is a ligand for Notch. Notch activation was scored using a reporter cell line in which H2B-YFP expression in the CHO-K1 cell nucleus induced by Notch activation is detected by fluorescence imaging^34^. On the control surface coated with ProG-TGT alone, we observed only background level of fluorescence. Strong nuclear fluorescence of YFP was detected on both 12 pN and 54 pN TGT surfaces (Fig. 5B), suggesting that the activity of DLL-1 is maintained after surface immobilization through ProG-TGT and that the molecular tension required for Notch activation is lower than 12 pN as we previously reported^13^.

## Conclusion

In summary, we designed and synthesized ProG-TGT for the study of molecular tensions on mechano-sensitive receptors. The general strategy of direct conjugation of a ligand to a TGT or to other force-reporting tethers is not ideal for large protein ligands because the procedures used for conjugation, purification and storage may greatly reduce the ligand’s activity which was the case in our initial attempt to link Ecad directly to a DNA strand in a TGT. The new strategy of indirect binding of protein ligands to TGT through protein G was successful in maintaining the ligand activity for Ecad, P-selectin and Notch. For bulky TGTs containing Ecad and ProG, we found that the size of the spacer between a biotin and TGT is important, and longer spacers are necessary in order to avoid steric hindrance in ligand-TGT immobilization. Applying this approach to Ecad, we found that Ecad tension is lower than 12 pN during cadherin-mediated cell adhesion, but after stable cell adhesion, some cadherin tensions can rupture TGT with *T*_tol_ = 43 pN, revealing the existence of multiple cadherin tension levels, reminiscent of a previous Ecad study in which multi-level tensions were reported^30^. We validated this force measurement strategy by applying ProG-TGT to confirm the 40 pN peak tension across single integrin-ligand bonds during integrin-mediated cell adhesion. We have also tested ProG-based TGT’s applications to the studies of P-selectin and Notch receptor and presented preliminary data showing that the approach works. Our approach is simple and modular and should be applicable to many other receptor-ligand systems where the ligand can be recombinantly fused to an Fc domain. This strategy can also be adopted by other molecular force sensors such as PEG or peptide-based FRET sensors recently developed in the field^9^,^10^,^11^,^12^.

## Material and Methods

### ProG-TGT Synthesis

ProG-TGT was created by hybridizing ProG-ssDNA and complementary ssDNA-biotin with the biotin location determining the tension tolerance of the dsDNA tether. ProG-ssDNA and ssDNA-biotin synthesis protocols are as follows.

### ProG-ssDNA conjugation

ProG (ab49807, Abcam. Recombinant ProG with 6-His tag for purification). Single-stranded DNA (ssDNA) was purchased from Integrated DNA technologies, Inc. The sequence and modifications are shown below: 5-/5Cy3/GGC CCG CAG CGA CCA CCC/3ThioMC3-D/ -3.

1. Add 5 μL × [50 mM TCEP (Tris(2-carboxyethyl)phosphine hydrochloride, reduction reagent, catalog#: 20490, Thermo Scientific) + 50 mM EDTA] (in PBS, PH7.2~7.4) into 20 μL × 1 mM thiol modified DNA in PBS. React for 30 mins at room temperature. Purpose: To deprotect thiol group by cleaving ThioMC3-D and make thiol group available for thiol-maleimide reaction.
2. Purify the DNA using Bio-spin 6 column (732–6200, Bio-rad. Buffer exchanged by PBS). Immediately add 1.5 μL × 23 mM sulfo-SMCC (22122, Thermo Scientific, pre-dissolved in pure water) into the DNA solution. React for 1 min.
3. Add the DNA solution into 10 μL × 5 mg/ml ProG (Buffer exchanged by DPBS). React overnight in 4 °C.
4. Purify the product using Dynabead (10103D, life technologies) through his-tag purification. Buffer exchange the final reagent with DPBS using Bio-spin 6 column.

Typical reading of final product: 100 μL × 19 μM DNA and 400 μg/ml (15 μM) ProG.

### Preparation of complementary ssDNA-biotin

The complementary ssDNAs were ordered from Integrated DNA technologies with customized sequence and modifications:

*T*_tol_ = 12 pN after hybridization with ProG-ssDNA: 5-/5Biosg/-GGG TGG TCG CTG CGG GCC3

*T*_tol_ = 23 pN after hybridization with ProG-ssDNA: 5-GGG/iBiodT/GG TCG CTG CGG GCC-3

*T*_tol_ = 33 pN after hybridization with ProG-ssDNA: 5-GGG TGG/iBiodT/CG CTG CGG GCC-3

*T*_tol_ = 43 pN after hybridization with ProG-ssDNA: 5-GGG TGG TCG C/iBiodT/G CGG GCC-3

*T*_tol_ = 54 pN after hybridization with ProG-ssDNA: 5-GGG TGG TCG CTG CGG GCC/3Bio/-3

The biotin linker from the company is PEG2 with about 1 nm length.

To create TGT with 6 nm biotin linker, we ordered ssDNAs with Amine modification at various locations:

*T*_tol_ = 12 pN after hybridization with ProG-ssDNA: 5-/5AmMC6/-GGG TGG TCG CTG CGG GCC3

*T*_tol_ = 23 pN after hybridization with ProG-ssDNA: 5-GGG/iAmMC6T/GG TCG CTG CGG GCC-3

*T*_tol_ = 33 pN after hybridization with ProG-ssDNA: 5-GGG TGG/iAmMC6T/CG CTG CGG GCC-3

*T*_tol_ = 43 pN after hybridization with ProG-ssDNA: 5-GGG TGG TCG C/iAmMC6T/G CGG GCC-3

*T*_tol_ = 54 pN after hybridization with ProG-ssDNA: 5-GGG TGG TCG CTG CGG GCC/3AmMO/-3

The PEG12-biotin tag was conjugated to these ssDNAs through NHS ester-amine reaction using this protocol: Add 5 μL × 2 mM ssDNA in H_2_O and 5 μL × 250 mM NHS-PEG12-biotin (21312, Thermo Scientific) in DMSO to 100 μL × 0.1 M sodium tetraborate, pH 8.5 labeling buffer. React for 5 hours at 4 °C. Purify the ssDNA-PEG12-biotin through ethanol precipitation.

### Preparation of ProG-TGT

Mix ProG-ssDNA with complementary ssDNA-biotin. The mixing molar ratio is 1.1:1. Incubate the mixture at 4 °C overnight. Five ProG-TGT constructs with *T*_tol_ = 12~54 pN, respectively, were ready for use.

### Preparation of Ligand-Fc:ProG-TGT

ProG-TGT was mixed with ligand with Fc fusion at 1:1 molar ratio and incubated at room temperature for 15 min. The final construct ligand-Fc:ProG-TGT was thereby assembled.

### E-Cadherin-ssDNA conjugation

Note that this conjugation and purification process significantly reduced E-cadherin activity. Therefore direct conjugation between E-cadherin and ssDNA is not recommended. E-Cadherin (648-EC-100, R&D systems. Recombinant cadherin with 6-His tag for purification). Single-stranded DNA (ssDNA) was purchased from Integrated DNA technologies, Inc. The sequence and modifications are shown below: 5-/5Cy3/GGC CCG CAG CGA CCA CCC/3ThioMC3-D/-3

1. Add 10 μL × [50 mM TCEP + 50 mM EDTA] (in PBS, PH7.2~7.4) into 25 μL × 2 mM DNA in PBS. React for 30 mins.
2. Purify the DNA with PBS buffer exchanged Bio-spin 6 (732–6200, Bio-rad). Immediately add 2 μL × 23 mM sulfo-SMCC (22122, Thermo Scientific, pre-dissolved in pure water). React for 1 min.
3. Add all DNA into 25ul × 2mg/ml E-Cad protein (dissolved in H_2_O, Buffer exchanged by DPBS). React overnight in 4C.
4. Purified by dynabead for his-tag purification. Buffer exchange the final reagent with DPBS with Zeba™ Spin Desalting Columns, 7K MWCO

Yield: 160 μL 6.4 μM DNA + 250 μg/ml (2.3 μM) E-Cadherin in a typical conjugation.

### Human IgG-RGDfK conjugation

Add 5 μL × [50 mM TCEP + 50 mM EDTA] (in PBS, PH7.2~7.4) into 90 μL × 10 mg/mL Human IgG (I4506-10MG, Sigma-Aldrich) in PBS. React for 30 min at room temperature. Purpose: Cleave disulfide bonds of proteins into thiol groups for subsequent conjugation.

1. Add 20 μL × 23 mM sulfo-SMCC (22122, Thermo Scientific, pre-dissolved in pure water) into 100 μL × 11 mM RGDfK-NH_2_. React for 30 min at room temperature.
2. Mix solutions prepared at step 1 and 2 together and react for 1 hour at room temperature or overnight at 4 °C.
3. Purify the Human IgG-RGDfK from unreacted RGDfK (small molecules with MW less than 1 kDa) using desalting column Biospin 6 (732–6200, Bio-rad) which is buffer exchanged with PBS in advance.

### Ligand-Fc:ProG-TGT immobilization on pegylated glass surface

A glass coverslip was pegylated as described previously^35^,^36^. 5% PEG molecules on the surface are modified with biotin tags. Briefly, a pre-cleaned glass coverslip slide was aminosilanized and the amines reacted with the N-hydroxysuccinimide (NHS) ester–modified PEG, mPEG-SVA (MW 5,000, Laysan Bio, Inc), mixed with 5% of biotin-PEG-SVA (MW 5,000, Laysan Bio, Inc). To immobilize TGT through a biotin-neutravidin bond on a pegylated glass surface, 200 μg/ml neutravidin (31000, Thermo Fisher Scientific Inc.) in PBS was incubated on such a surface for 20 min. The coverslip was washed by PBS twice and dried by tilting it and allowing PBS solution to roll away from the surface (PEG surface is non-wetting to water and PBS). 5 μL droplets of ligand-Fc:ProG-TGTs were loaded on the neutravidin-coated surface with a pipette. This surface was incubated at 4 °C for 30 min and then washed by copious amounts of PBS solution. During this round of washing, the surface should always be submerged in PBS. Surface drying may increase the non-specific physical adsorption of protein ligand-TGT on the surface. The PBS will be exchanged with cell culture medium during the cell plating procedure.

### Preparation of cell solution

All adherent cells were detached from culture flasks using a mild detaching reagent, EDTA (Ethylenediaminetetraacetic acid), solution to preserve the integrity of cell membrane proteins. Cells in flasks were rinsed by the EDTA solution three times, incubated at 37 °C in the EDTA for 15 min and dispersed by pipetting. Cells were spun down at a 300 × g centrifuging rate and re-suspended in serum-free medium at 10^6^/mL for force measurement (RPMI medium for DLD-1 cells and αMEM medium for CHO-K1 cells). The recipe for the EDTA solution is: 100 mL 10 × HBSS + 10 mL × 1 M HEPES (PH 7.6) + 10 mL × 7.5% sodium bicarbonate + 2.4 mL × 500 mM EDTA + 1 L H_2_O.

### CHO-K1 cell plating and incubation on human IgG-RGDfK-TGT surfaces

Prepared CHO-K1 cell solution was plated on TGT surfaces and incubated in 37 °C and 5% CO_2_ for 30 min. Non-adherent cells were removed by gentle pipetting and buffer exchange. Note that the PEG surface must be always kept in medium because exposure to air would detach the adherent cells from the non-wetting pegylated surface. The adherent cells were imaged and cell density was quantified.

### DLD-1 cell plating and incubation on cadherin-TGT surfaces

Prepared DLD-1 cell solution was plated on the cadherin-TGT surface and incubated in 37 °C and 5% CO_2_ for 2 hours. Note that DLD-1 cell incubation requires a longer time because we observed that cadherin-mediated cell adhesion is generally slower than integrin-mediated cell adhesion. Non-adherent cells were removed by gentle pipetting and buffer exchange.

### Notch receptor activation

To test the molecular tension range required by Notch activation, we immobilized Notch ligands through TGTs on a glass surface. Delta-like ligand (DLL1) with Fc fusion was mixed with ProG-TGT at 1:1 molar ratio in PBS for 30 min at 0.1 μM concentration. A glass-bottom petridish was incubated with a mixture of 50 μg/ml BSA-biotin (bovine serum albumin with biotin tags) and 50 μg/ml fibronectin for 1 hour. Fibronectin helps cells to adhere. The glass surface was then incubated with 200 μg/mL neutravidin for 30 min and washed by PBS. Subsequently 0.1 μM DLL1-Fc:ProG-TGT construct was incubated on such a surface for 30 min and the construct was immobilized through biotin-neutravidin interaction. On the surface we seeded CHO-K1 cells which stably express human NOTCH1 whose intracellular domain is replaced by the activator Gal4^esn^. This cell is also transfected with genes of UAS controlled H2B–YFP as reporter. After notch activation, Gal4^esn^ is released and binds to UAS and then activates the H2B-YFP expression in nucleus. Cell seeding density is 0.5 × 10^5^/mL. The YFP expression reaches an optimal level after two days.

### Leukocyte rolling test

Leukocytes express P-selectin glycoprotein ligand (PSGL-1) on their cell membrane. To study leukocyte rolling mediated by P-selectin and PSGL-1 bonds, we immobilized P-selectin through TGTs on a pegylated glass surface. P-selectin with Fc fusion (137-PS-050, R&D systems) was mixed with ProG-TGT at 1:1 molar ratio in PBS for 30 min at 0.25 μM concentration. A flow chamber with one pegylated inner glass surface was prepared. The glass surface was functionalized with PEG polymer (5% PEG was tagged with biotin). This flow chamber was incubated with 200 μg/mL neutravidin for 30 min and washed by PBS. Subsequently 0.1 μM of P-selectin-Fc:ProG-TGT construct was incubated in the flow chamber for 30 min and the construct was immobilized through biotin-neutravidin interaction. Leukocytes (HL-60 cells) were flowed through the chamber at a flow rate 50 μm/s. The attachment and rolling of leukocytes was captured by time-trace live cell imaging.

## Additional Information

**How to cite this article**: Wang, X. *et al.* Constructing modular and universal single molecule tension sensor using protein G to study mechano-sensitive receptors. *Sci. Rep.* **6**, 21584; doi: 10.1038/srep21584 (2016).

## Supplementary Material

Supplementary Information
