# Supplementary material for: Constructing modular and universal single molecule tension sensor using protein G to study mechano-sensitive receptors
Source: Sci Rep. 2016 Feb 15;6:21584. doi: 10.1038/srep21584 (PMC4753514; doi:10.1038/srep21584)
Supplement: Supplementary Information [file srep21584-s1.doc]

**Supplementary Materials**

**Constructing modular and universal single molecule tension senor using protein G to study mechano-sensitive receptors**

Xuefeng Wang^a,b,c^, Zainab Rahil^a,d^, Isaac T. S. Li^a^, Farhan Chowdhury^b,e^, Deborah Leckband^d^, Yann Chemla^a^ and Taekjip Ha^a,b,f,g^*

^a^Department of Physics, Center for the Physics of Living Cells, University of Illinois at Urbana-Champaign, Urbana, Illinois 61801, USA;

^b^Institute for Genomic Biology, University of Illinois at Urbana-Champaign, Urbana, Illinois 61801, USA;

^c^Department of Physics and Astronomy, Iowa State University, Ames, Iowa 50011, USA;

^d^Department of Chemistry, University of Illinois at Urbana-Champaign, Urbana, Illinois 61801, USA;

^e^Mechanical Engineering and Energy Processes, Southern Illinois University, Carbondale, Illinois 62901,USA

^f^Howard Hughes Medical Institute, Urbana, IL 61801, USA

^g^Department of Biophysics & Biophysical Chemistry, Department of Biophysics & Department of Biomedical Engineering, Johns Hopkins University, Baltimore, MD 21205, USA

*Corresponding author. E-mail: [tjha@jhu.edu](mailto:tjha@jhu.edu)

**Verification of Ecad activity after binding to Protein G**

To confirm that Ecad activity is not reduced after binding to protein G (ProG), we mixed Ecad and ProG in a molar ratio 1:1 at 1 µM and incubated the mixture in room temperature for 30 min. A glass bottom petridish was coated by Ecad:ProG for 30 min and blocked with 200µg/ml bovine serum albumin for 1 hour. With the same procedure, we also prepared Ecad and Ecad-ssDNA coated glass bottom petridishes. DLD-1 cells detached by EDTA solution were seeded on three surfaces and incubated for 2 hours. Cells adhered and spread normally on surfaces physically adsorbed with Ecad:ProG or Ecad, but cell adhesion and spreading was compromised on Ecad-ssDNA coated surface. This experiment verified that Ecad activity was not reduced due to binding with ProG, but significantly reduced due to the procedure of ssDNA conjugation.


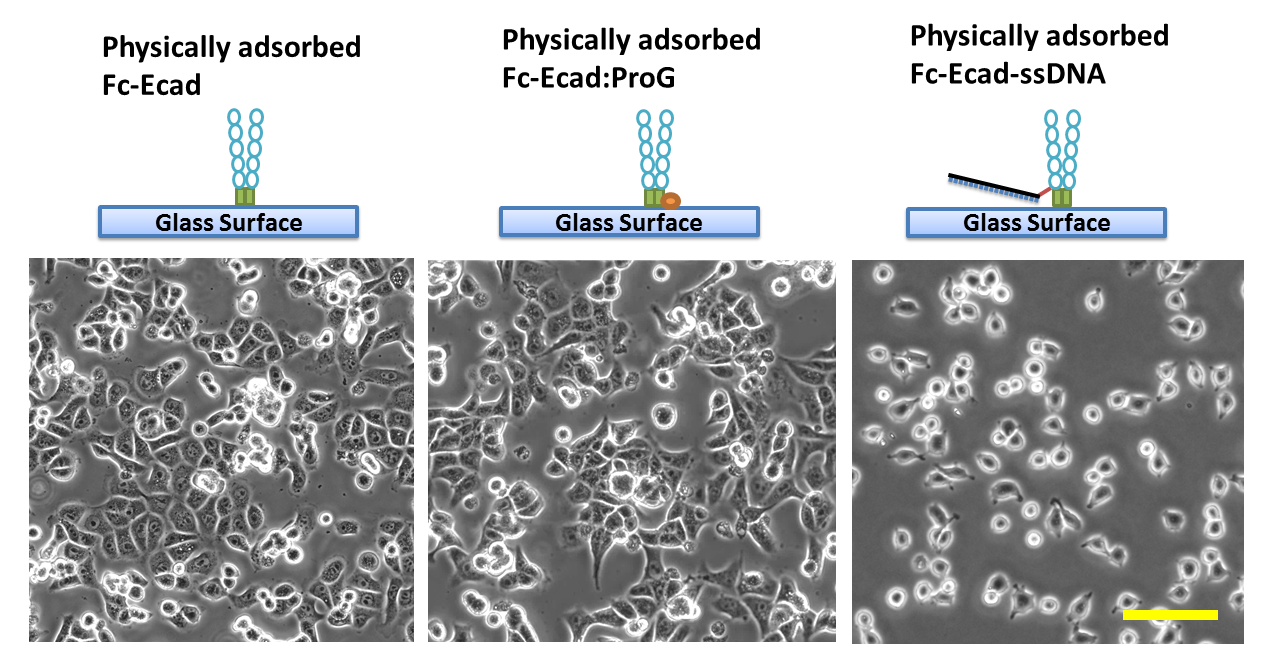


**SFig. 1** DLD-1 cell adhesion on Ecad-Fc, Ecad-Fc:ProG and Ecad-Fc-ssDNA coated surfaces. Scale bar: 100 µm

**Ecad activity is retained after being immobilized on PEG surface through ProG-biotin**

To confirm that Ecad activity is not lost after being immobilized through ProG-biotin on pegylated glass surface which is the main experimental platform we used in this article, we tested DLD-1 cell adhesion on Ecad-Fc:ProG-biotin:neutravidin:bio-PEG surface on which we successively incubated with 200 µg/ml neutravidin and the premixed solution of Ecad-Fc:ProG-biotin at 1µM. DLD-1 cells adhered and spread normally on such surface but did not adhere or spread well on ProG-biotin:neutravidin:bio-PEG surface, indicating that Ecad activity was not lost after immobilization on pegylated glass and cell adhesion was mediated by Ecad, not ProG.


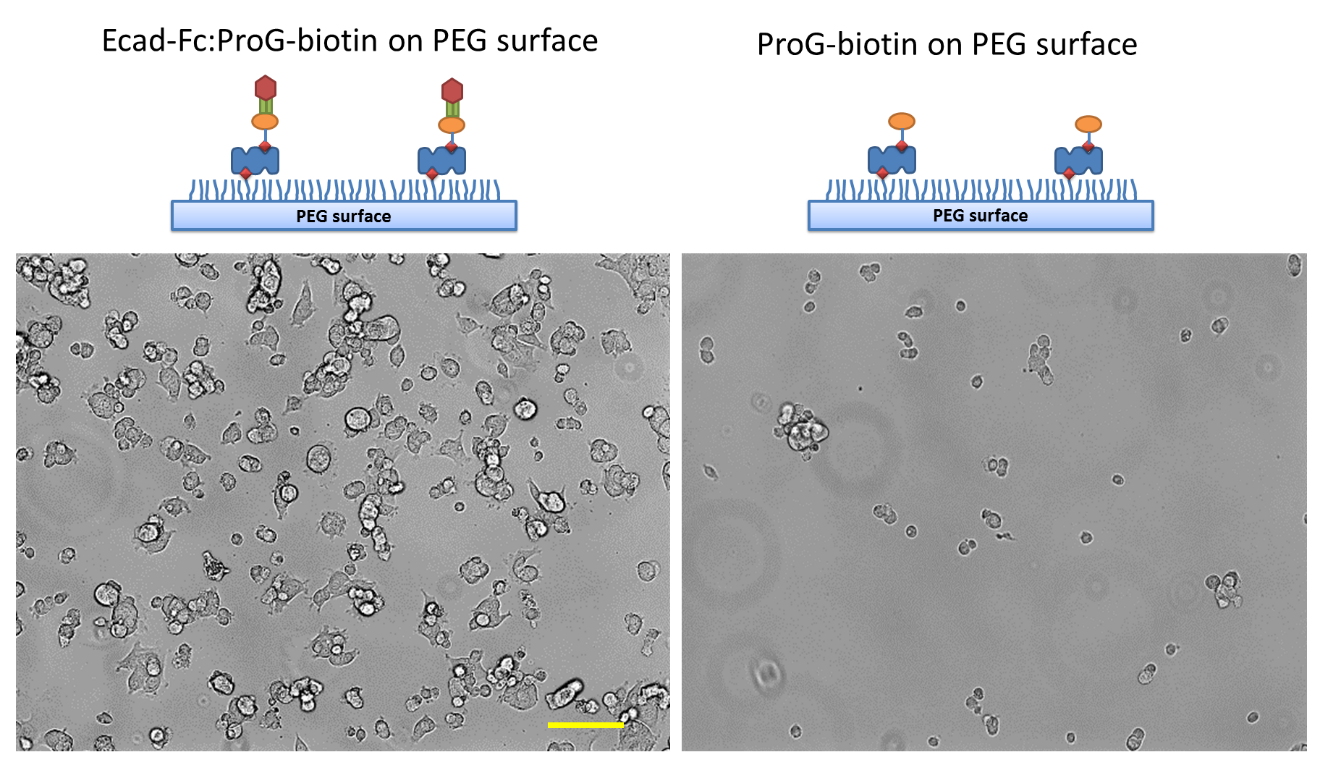


**SFig. 2** DLD-1 cells adhere and spread well on Ecad-Fc:ProG-biotin:neutravidin:bio-PEG surface. DLD-1 cells did not adhere or spread well on ProG-biotin:neutravidin:bio-PEG surface. Scale bar: 100 µm

**6 nm long PEG12 linker used for TGT immobilization to overcome the steric hindrance caused by large-sized ligand molecules.**

Due to the large size of the final Ecad-TGT construct (Overall MW: 211 kDa), steric hindrance became an issue when we used a short spacer (PEG2, Integrated DNA Technologies) for biotin attachment to TGT (SFig. 3A). PEG2, which is a commonly used spacer for biotin modification on DNA and was used in an earlier TGT work , gave uneven surface density of immobilized ligands (SFig. 3B). After TGT incubation at a saturated concentration (1 µM), the surface density of 12, 23 and 33 pN TGT was only half the density of 54 pN TGT, likely because of relative proximity between biotin and ProG for TGTs with lower *T*_tol_ values that caused greater steric hindrance (SFig. 3B). The coefficient of variation of ligand density across the five TGT surfaces was 0.33. When we switched to PEG12 spacer with 6 nm contour length, we could obtain much more even surface densities for all TGTs with a much reduced coefficient of variation of 0.04 (SFig. 3), presumably because the biotin with a 6 nm spacer has a longer reach to neutravidin on the surface, therefore compensating for the steric hindrance caused by the large size of ligand-ProG. In all remaining data presented, we used the PEG12 spacer.


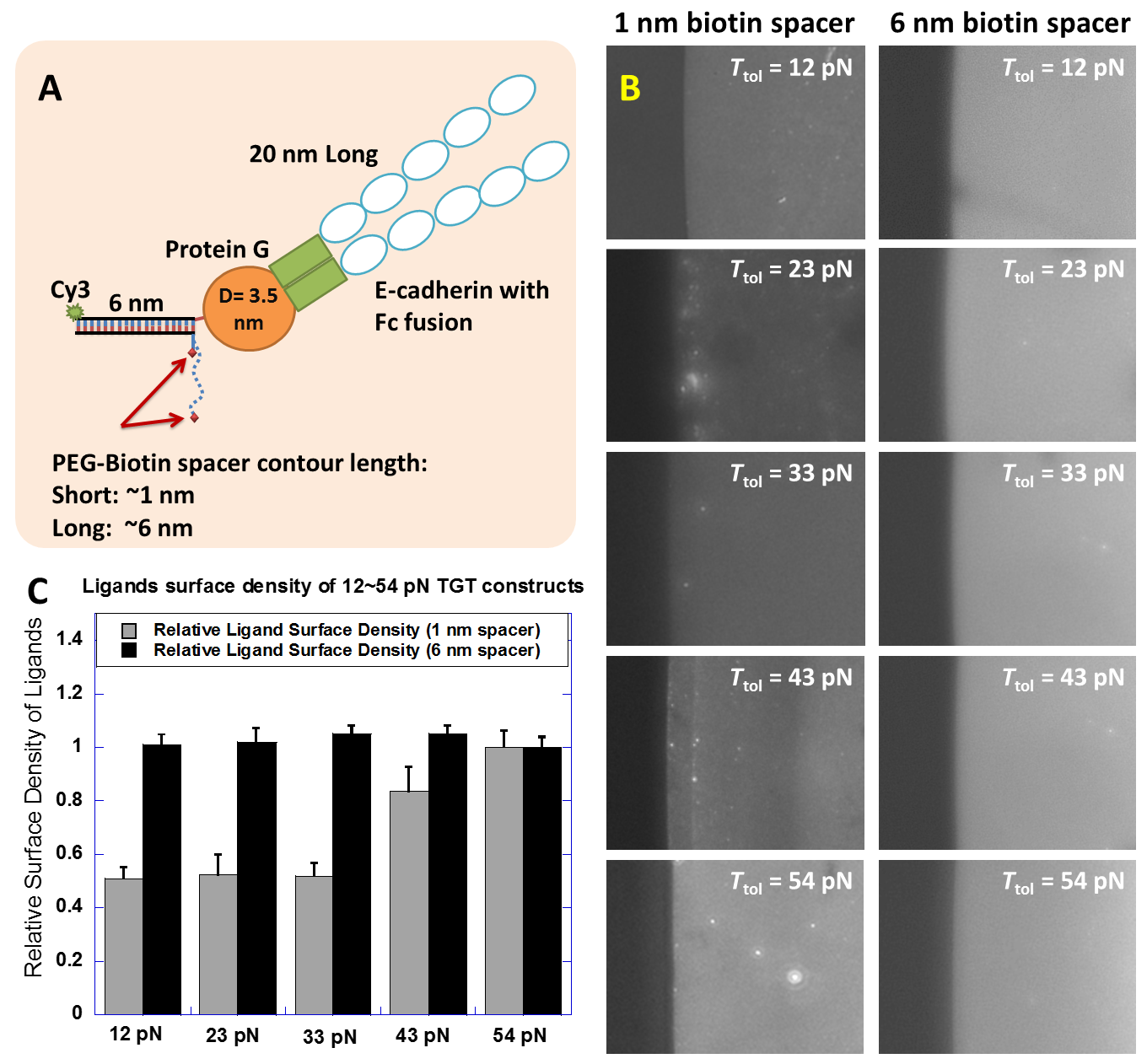


**SFig. 3** 6 nm biotin-spacer minimizes steric hindrance. (A) Schematics of Ecad-Fc:ProG-TGT complex. (B) Fluorescence images of regions coated with 12~54 pN ProG-TGTs through 1 nm or 6 nm biotin spacers. (C) ProG-TGT surface densities reported by fluorescence intensities. Coefficient of variation (standard-deviation-to mean ratio) of surface densities of 12~54 pN TGTs with 1 nm biotin-spacer is 0.33. For TGTs with 6 nm biotin spacer, coefficient of variation is 0.04.
